# A Cost-Effective Silica Fume Coating Layer for Stable Zn Metal Anodes

**DOI:** 10.3390/ma19051000

**Published:** 2026-03-05

**Authors:** Yuxing Zhang, Jiaxuan Cheng, Pan Chen, Yuxin Zhao, Yuhan Wang, Yuanming Shi, Jihua Zhai

**Affiliations:** College of Materials Science and Engineering, Sichuan University, Chengdu 610065, China

**Keywords:** aqueous zinc-ion batteries, zinc anode, zinc dendrites, coating layer, silica fume

## Abstract

**Highlights:**

**What are the main findings?**

- SF@Zn can promote uniform deposition between silica particles and suppress dendrite growth.
- The symmetric cell with SF@Zn achieved stable cycling for 1800 h at 0.5 mA·cm^−2^, while the full cell delivered a capacity of 246.2 mAh·g^−1^ at 1.0 A·g^−1^ and retained a capacity of 100.4 mAh·g^−1^ after 1800 cycles.

**What are the implications of the main findings?**

- The use of silica fume as a protective layer represents a high-value application of industrial byproducts.
- The use of silica fume as a protective layer offers new insights into anode design for aqueous zinc-ion batteries.

**Abstract:**

Aqueous zinc-ion batteries have emerged as a research hotspot due to their advantages of safety, environmental friendliness, low cost, and high capacity. At the same time, there are some problems with anode materials, such as zinc dendrite growth and corrosion reactions. In this work, silica fume, a byproduct of industrial silicon smelting, was selected as a coating material for the Zn anode (SF@Zn). This material is not only cost-effective and widely available but also exhibits superior hydrophilicity, enhancing the electrolyte’s wettability on the anode. Additionally, it serves as an ion shunt, preventing uneven deposition of Zn^2+^, and it was demonstrated that the symmetrical cell achieved a cycle life of up to 1800 h at 0.5 mA·cm^−2^. The full cell delivered a capacity of 246.2 mAh·g^−1^ at 1 mA·cm^−2^ and retained a capacity of 100.4 mAh·g^−1^ after 1800 cycles.

## 1. Introduction

In recent years, with the rapid development of the economy and industry, the demand for energy storage systems has continued to grow [1,2,3]. Although traditional lithium-ion batteries are widely used in electronics and automotive applications, their organic electrolytes pose safety risks due to toxicity and flammability, limiting their further market expansion [4,5,6,7,8]. Consequently, there is an urgent need for battery systems with high capacity, low cost, and enhanced safety. Aqueous metal-ion batteries, with their high ionic conductivity, non-flammability, and cost-effectiveness, are considered the most promising alternatives to lithium-ion batteries [9,10,11,12,13].

Among them, aqueous zinc-ion batteries have attracted significant attention due to their high theoretical specific capacity (820 mAh/g), volumetric capacity (5855 mAh/cm^3^), and low equilibrium potential [14,15,16,17]. However, inherent protrusions on the zinc foil surface can lead to localized electric field enhancement, resulting in uneven Zn^2+^ concentration distribution during subsequent ion diffusion. Areas with higher concentrations tend to deposit more Zn^2+^, forming dendrites [18,19,20]. The continuous growth of zinc dendrites can penetrate the separator, causing internal short circuits. Additionally, issues such as hydrogen evolution reactions, corrosion, and passivation further degrade the performance of aqueous zinc-ion batteries, hindering their industrialization [21,22,23,24].

Addressing these anode-related issues is crucial for improving the overall electrochemical performance of the batteries. Current strategies include electrolyte modification, anode structural design, and artificial interface layer construction [25,26,27,28,29,30,31]. These approaches enhance the electrochemical stability of the anode through the following mechanisms: (1) reducing the number of active water molecules at the anode/electrolyte interface to suppress corrosion reactions [32,33]; (2) establishing a uniform electric field to regulate Zn^2+^ deposition and prevent uneven deposition [34,35]; and (3) constructing an interface layer to provide abundant and uniform nucleation sites, inhibiting zinc dendrite growth [36,37]. Among these methods, constructing an artificial interface layer is one of the most effective strategies as it not only suppresses dendrite formation but also protects the anode surface from corrosion. Common interface layers include metal/non-metal oxide coatings (e.g., CaCO_3_ [38], Al_2_O_3_ [39], TiO_2_ [40], ZrO_2_ [41], Sc_2_O_3_ [42], ZnS [43], and BaTiO_3_ [44]), organic coatings (e.g., polyamide [45] and ethylene polymers [46]), and two-dimensional carbon/MXene-based coatings (e.g., graphite [36], graphene [47], and carbon nitride [48]).

Metal/non-metal oxide coatings are typically applied to the zinc anode surface via physical methods, preventing direct contact between the anode and electrolyte to mitigate hydrogen evolution reactions. Depending on the material, these coatings can exhibit varying degrees of zincophilicity, regulating Zn^2+^ flux and alleviating dendrite formation. For instance, Kang et al. [38] designed a nanoporous calcium carbonate coating that modulates the electrolyte flux on the zinc surface, influencing the deposition rate and promoting uniform, bottom-up deposition to avoid large dendrites. Liang et al. [41] employed a sol–gel method to prepare nano-zirconia as an inert coating on the zinc anode, physically isolating the electrode from the electrolyte to suppress hydrogen evolution and reduce anode polarization. In such studies, coating materials are often synthesized directly from chemical precursors, increasing costs and complicating industrial scalability. Zhong et al. [49] used superhydrophobic SiO_2_ nanoparticles with an average size of approximately 28.4 nm as the coating. The cell operated over 1500 h at 0.1 mA cm^−2^.

In contrast, we chose silica fume. Silica fume is an ultrafine siliceous powder formed during the smelting of ferrosilicon and industrial silicon, where SiO_2_ and Si gases rapidly oxidize and condense in air, forming amorphous spherical particles with an average diameter of 0.1–0.3 μm. Traditionally used as an additive in construction materials, silica fume is abundant and low-cost and exhibits high specific surface area due to its spherical particles, making it an ideal dense protective layer material. This study presents a novel approach to the resource utilization of silica fume in aqueous zinc-ion batteries.

In this work, silica fume was employed as a protective layer material for the zinc anode in aqueous zinc-ion batteries. The silica fume consists of SiO_2_, with the remainder being impurities such as Fe, K, Ca, and Al, along with trace insoluble impurities. These impurities can affect the coating uniformity on the anode surface and interfere with Zn^2+^ redox reactions. Therefore, pretreatment is necessary to reduce impurity content and ensure battery performance. The silica fume protective layer primarily functions by physically isolating the electrode from active water molecules in the electrolyte, guiding uniform Zn^2+^ deposition. Experimental results demonstrate that the silica fume-coated zinc anode (SF@Zn) exhibits more stable operating voltage and longer cycle life compared to the bare zinc anode.

## 2. Experimental

### 2.1. Material Preparation

#### 2.1.1. Cathode Material

Sodium vanadate (Aladdin, Shanghai, China) (1.264 g) was dissolved in 74 mL of deionized water under stirring at 80 °C. The solution was then transferred to a magnetic stirrer, and 200 drops of dilute hydrochloric acid (Aladdin, Shanghai, China) (3 mol/L) were added dropwise. Subsequently, 46 mL of methanol (Aladdin, Shanghai, China) was introduced, and the mixture was stirred for 10 min before being transferred to an autoclave and heated at 170 °C for 12 h. The product was filtered, washed, dried, and calcined at 350 °C in air for 2 h to obtain vanadium pentoxide (V_2_O_5_) as the cathode material. The V_2_O_5_, carbon black, and PVDF (Aladdin, Shanghai, China) were mixed in a weight ratio of 7:2:1 and coated onto titanium foil, followed by drying at 80 °C for 6 h.

#### 2.1.2. Silica Fume Pretreatment

Untreated silica fume (100 g) was repeatedly ground and sieved through a 400-mesh sieve. It was then dissolved in deionized water and left to stand for 1 h to remove floating impurities. Magnetic impurities containing Fe were removed using a strong magnet. Next, 125 mL of 2 M HCl was added dropwise under stirring for 2 h, followed by filtration and washing with deionized water three times. The treated silica fume was dried at 80 °C for 6 h and mixed with PVDF in a weight ratio of 9:1 before being coated onto zinc foil.

### 2.2. Electrochemical Measurements

For Zn-Zn symmetric cells, zinc foil or silica fume-coated zinc foil (SF@Zn) was used as the electrode, with a glass fiber separator (18 mm diameter) and 2 M ZnSO_4_ solution as the electrolyte. For SF@Zn-V_2_O_5_ full cells, SF@Zn served as the anode, V_2_O_5_ as the cathode, and the same separator and electrolyte were used. Galvanostatic charge/discharge (GCD) tests were carried out on a CT4008Tn battery testing system. Cyclic voltammetry (CV) and electrochemical impedance spectroscopy (EIS) measurements were performed on a PARSTAT4000A electrochemical workstation (Ametek, Berwyn, PA, USA).

### 2.3. Characterization Techniques

The electrode material and electrodes were characterized by a Rigaku-SmartLab X-ray diffractometer (XRD) (Rigaku, Tokyo, Japan), a Rigaku-ZSX Primus III NEXT X-ray Fluorescence Spectrometer (XRF) (Rigaku, Tokyo, Japan), Malvern Panalytical-Zetasizer Pro Nanometrics (DLS) (Malvern Panalytical Ltd., Malvern, UK), a ZEISS-Sigma 360 field emission Scanning Electron Microscope (SEM) (ZEISS, Oberkochen, Germany), and a Dataphysics-OCA 20 contact angles analyzer (Dataphysics, Filderstadt, Germany).

## 3. Results and Discussion

### 3.1. Characterization of Silica Fume

The silica fume employed in this study primarily consists of amorphous SiO_2_, with additional components including Fe_2_O_3_, K_2_O, CaO, Al_2_O_3_, and trace insoluble impurities. Direct application of untreated silica fume in zinc-ion batteries would introduce multiple detrimental effects: (1) metallic impurities (particularly Fe_2_O_3_) may participate in redox reactions, impeding charge transfer at the anode interface; (2) insoluble contaminants would compromise coating uniformity, resulting in incomplete surface coverage and loss of protective functionality.

**Figure 1:**
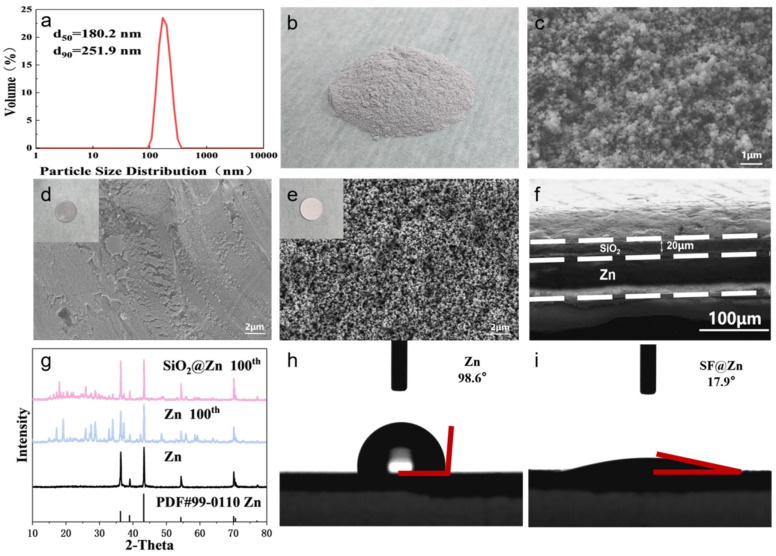
(**a**) DLS patterns of silica fume. (**b**) Photograph of silica fume. (**c**) SEM images of silica fume. Photographs and SEM images of (**d**) pure Zn and (**e**) SF@Zn. (**f**) Cross-sectional SEM image of SF@Zn. (**g**) XRD patterns of pure Zn and SF@Zn after 100 cycles. Contact angles of (**h**) pure Zn and (**i**) SF@Zn.

To address these challenges, we implemented a comprehensive purification protocol combining physical and chemical treatments. Table 1 shows the XRF results of the pretreated silica fume. The purified material presented as grayish-white spherical particles with an average diameter of approximately 250 nm (Figure 1a–c), a morphological characteristic resulting from rapid condensation during the industrial production process.

The nanoscale particle size provides two critical advantages for battery applications: (1) the substantially increased specific surface area (BET surface area > 20 m^2^/g) enhances interfacial contact between the protective layer and electrolyte, and (2) the spherical morphology enables dense packing on the zinc foil surface, creating uniform nanochannels for regulated Zn^2+^ flux. This optimized physical structure, combined with the chemical purity achieved through our purification process, establishes an ideal foundation for high-performance anode protection in aqueous zinc-ion batteries.

In aqueous zinc-ion batteries, unmodified zinc foil surfaces exhibit uneven protrusions, causing Zn^2+^ to preferentially deposit at these sites during charging/discharging, exacerbating dendrite growth. To optimize the electrode/electrolyte interface and regulate Zn^2+^ deposition, pretreated silica fume was uniformly coated onto the zinc foil, forming a protective layer approximately 20 μm thick. The anode surface was evenly covered with spherical silica particles (Figure 1d–f). XRD analysis of the anode after 100 cycles at 1 mA·cm^−2^ revealed new peaks on the bare zinc foil, likely due to corrosion products such as (Zn(OH)_2_)_3_(ZnSO_4_)(H_2_O)_3_ [50]. In contrast, the silica fume-coated anode showed significantly fewer new peaks and lower peak intensities (Figure 1g), confirming the protective role of the coating. Contact angle tests further demonstrated that SF@Zn exhibited superior hydrophilicity compared to bare zinc foil, promoting better electrolyte–anode contact (Figure 1h–i).

### 3.2. Zn Plating/Striping Behavior in Anodes

**Figure 2:**
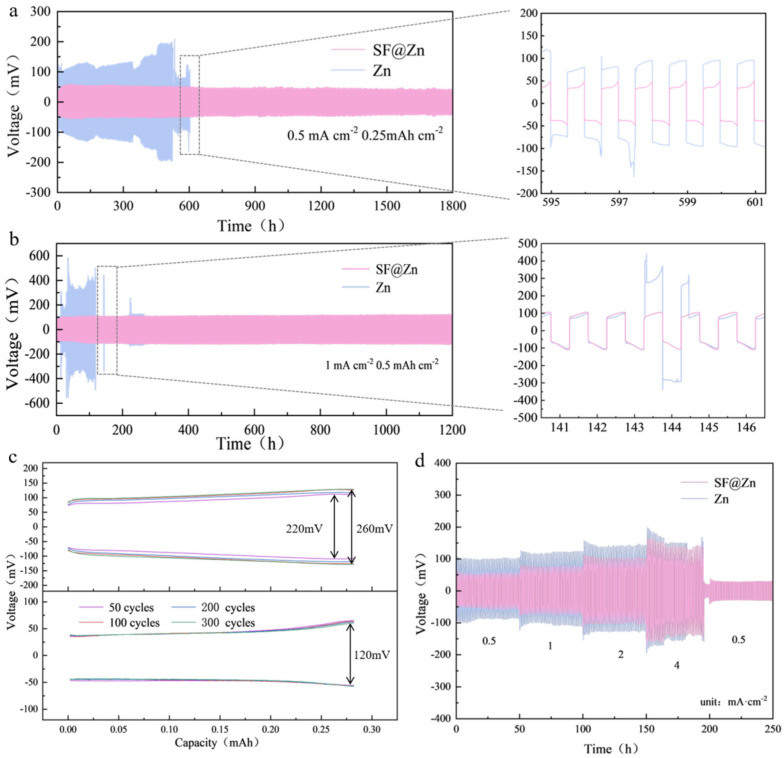
(**a**) Cycling performance at different current densities of (**a**) 0.5 mA·cm^−2^ and (**b**) 1.0 mA·cm^−2^. (**c**) Voltage profiles in different cycles at 0.5 mA·cm^−2^. (**d**) Rate performance at various current densities of 0.5, 1.0, 2.0, 4.0 and 0.5 mA·cm^−2^.

Galvanostatic cycling tests of symmetric cells at 0.5 and 1.0 mA·cm^−2^ were conducted to evaluate the impact of the silica fume coating. At 0.5 mA·cm^−2^, the bare zinc anode exhibited a cycle life of 600 h with significant voltage fluctuations, while SF@Zn achieved a stable cycle life of 1800 h (Figure 2a). The silica fume coating physically isolated active water molecules from the anode, improved the interface environment, and promoted uniform Zn^2+^ deposition, enhancing voltage stability and cycle life. At 1.0 mA·cm^−2^, SF@Zn maintained stable voltage for 1200 h, whereas the bare zinc anode showed a severe voltage drop after 121 h (Figure 2b), indicating irreversible redox reactions. Overpotential analysis revealed that the bare zinc anode increased from 110 mV at 50 cycles to 130 mV at 300 cycles, while SF@Zn maintained a stable overpotential of 60 mV even after 1800 cycles (Figure 2c). Rate performance tests (Figure 2d) further confirmed that SF@Zn exhibited lower voltage hysteresis and better stability across current densities of 0.5, 1, 2, and 4 mA·cm^−2^.

**Figure 3:**
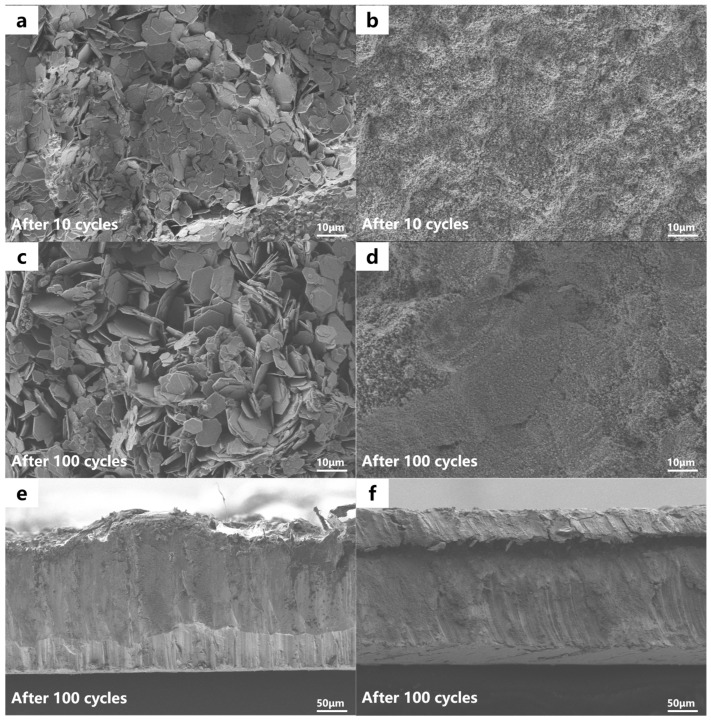
SEM images of (**a**,**c**) pure Zn and (**b**,**d**) SF@Zn after 10 and 100 cycles at 2.0 mA·cm^−2^ for symmetric battery. SEM images of cross-sectional (**e**) pure Zn and (**f**) SF@Zn after 100 cycles at 2.0 mA·cm^−2^ for symmetric battery.

To systematically evaluate the dendrite-inhibiting efficacy of the silica fume (SF) protective coating, we conducted comprehensive morphological characterization of anode surfaces following galvanostatic cycling at 2 mA·cm^−2^ for 10 and 100 cycles, respectively. As illustrated in Figure 3a, the unmodified zinc anode exhibited pronounced flake-like zinc deposition after merely 10 cycles, accompanied by substantial surface roughening. Progressive cycling exacerbated this phenomenon, with the anode surface becoming entirely occluded by enlarged dendritic structures after 100 cycles (Figure 3c). Uncontrolled dendrite propagation may lead to mechanical fracture during prolonged cycling, resulting in the formation of electrochemically inactive “dead zinc” that becomes detached from the current collector and subsequently dispersed within the electrolyte phase. Cross-sectional analysis provided further mechanistic insights, revealing two critical failure modes: (i) the presence of embedded glass fiber fragments (Figure 3e), indicative of dendrite penetration through the separator with consequent short-circuit risk, and (ii) the development of corrosion-induced surface pitting, which promotes deleterious side reactions and diminishes Coulombic efficiency. In striking contrast, the SF@Zn composite anode demonstrated exceptional morphological stability throughout cycling. Post-10-cycle examination revealed complete preservation of the original spherical silica particulate morphology (Figure 3b), with no detectable dendrite nucleation. This protective functionality remained robust even after extended cycling, with the anode surface maintaining a homogeneous and compact silica matrix after 100 cycles (Figure 3d). Cross-sectional electron microscopy confirmed the structural integrity of the SF coating layer, showing neither zinc dendrite penetration nor corrosion-induced topographical defects (Figure 3f). These results provide evidence that the silica fume interface layer can suppresses dendrite initiation and mitigate corrosion processes.

**Figure 4:**
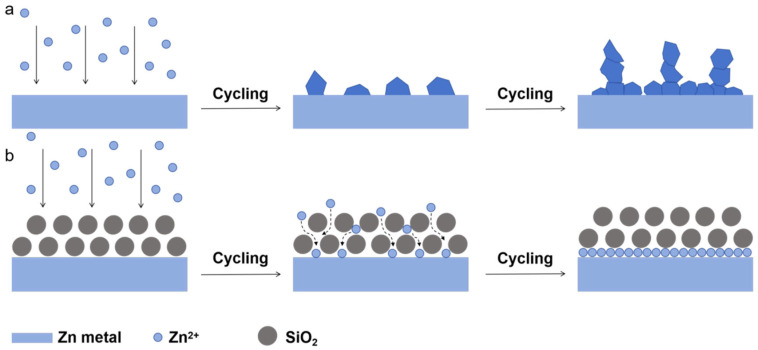
Schematic illustrations of (**a**) pure Zn foils and (**b**) SF@Zn in the cycle process.

Based on the experimental results, it can be concluded that the silica fume protective layer effectively prevents zinc dendrite growth and suppresses corrosion reactions. As illustrated in Figure 4, during Zn^2+^ deposition on the anode, inherent surface defects on bare zinc foil serve as preferential nucleation sites for Zn^2+^. Due to the tip effect, these sites facilitate rapid dendritic growth, leading to uneven deposition and eventual battery failure. In contrast, the SF coating significantly modifies the anode surface environment. The densely packed spherical silica particles create uniform interstitial spaces that guide homogeneous Zn^2+^ deposition, thereby preventing localized dendrite formation. Furthermore, the SF layer acts as a physical barrier, partially isolating the anode from active water molecules in the electrolyte. This dual functionality—(1) regulating ion flux to ensure uniform plating and (2) mitigating parasitic reactions—collectively enhances the overall electrochemical performance of the battery. These findings demonstrate that the silica fume coating not only addresses the fundamental challenges of zinc anodes but also provides a scalable and cost-effective strategy for developing high-performance aqueous zinc-ion batteries.

### 3.3. Electrochemical Performance of Full SF@Zn--V_2_O_5_ Cells

**Figure 5:**
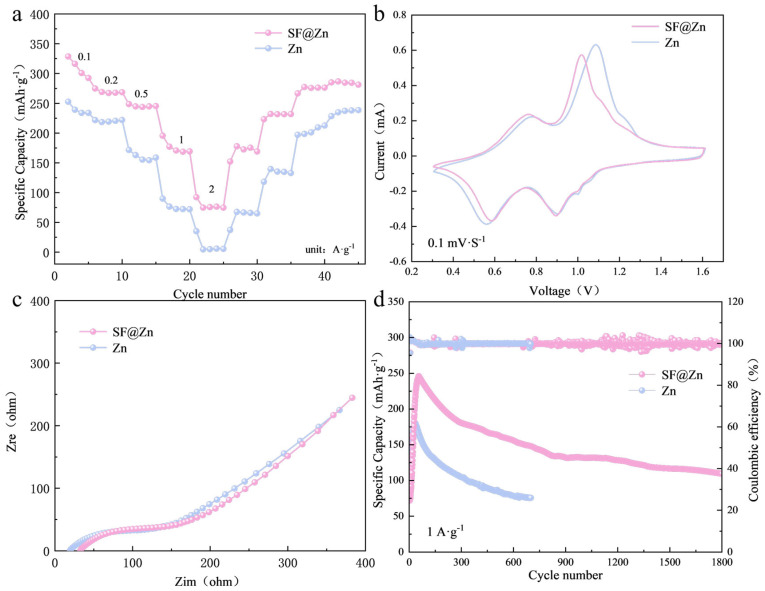
(**a**) Rate performance at the current densities of 0.1, 0.2, 0.5, 1.0 and 2.0 A·g^−1^. (**b**) CV curves in the potential range of 0.3–1.6 V with a scan rate of 0.1 mV·s^−1^. (**c**) EIS of V_2_O_5_-Zn cell and V_2_O_5_-SF@Zn cell. (**d**) Galvanostatic cycling performance at a current density of 1.0 A·g^−1^.

The practical performance of SF@Zn was evaluated in full cells with V_2_O_5_ cathodes. Figure 5a shows the rate performance of the two full cells. Notably, the SF@Zn||V_2_O_5_ cell delivers an absolutely higher specific capacity at all current densities. The CV curves of the cells with different anodes have the same shape in Figure 5b. In the SF@Zn||V_2_O_5_ cell, the reduction reactions from V^5+^ to V^4+^ and then to V^3+^ may correspond to the parks at 0.60 and 0.90 V. The anodic peaks at 0.78 V and 1.02 V correspond to reverse oxidation reactions. In Zn||V_2_O_5_ cell, the peak at 0.60 V changes to 0.56 V, and the peak at 1.02 V changes to 1.10 V. For an electrochemically reversible system, the peak potential difference (Δ*E_p_*) is a criterion for judging the reversibility of the electrode process.

$$\Delta E_{p}=\left|E_{pa}-E_{pc}\right|$$

Among them, *E_pa_* is the anodic peak potential and *E_pc_* is the cathodic peak potential. The Δ*E_p_* of the cell after coating modification is smaller, indicating that its reversibility is better.

Figure 5c shows the EIS plots of two cells. The semi-circle radii of the impedance diagrams of the two are similar to the slopes of the straight lines, indicating that the charge transfer impedance and diffusion impedance of the two are close. This suggests that the nano-silica protective layer has a relatively small impact on the conductivity of the battery. At 1.0 A·g^−1^, the SF@Zn cell delivered a capacity of 246.2 mAh·g^−1^ and retained 100.4 mAh·g^−1^ after 1800 cycles, representing a capacity retention of 40.8%, whereas the bare zinc cell achieved only 180.7 mAh·g^−1^ and degraded to 75.7 mAh·g^−1^ after 700 cycles, representing a capacity retention of 41.8% (Figure 5d). These results demonstrate that the silica fume coating effectively suppresses side reactions and extends battery lifespan.

## 4. Conclusions

In summary, silica fume was employed as a protective coating for the zinc anode in aqueous zinc-ion batteries. This cost-effective coating not only regulated Zn^2+^ deposition, promoting uniform deposition between silica particles and suppressing dendrite growth, but also physically isolated the anode from active water molecules, reducing corrosion. Consequently, the battery’s cycle life was significantly improved. The symmetric cell with SF@Zn achieved stable cycling for 1800 h at 0.5 mA·cm^−2^, while the full cell delivered a capacity of 246.2 mAh·g^−1^ at 1.0 A·g^−1^ and retained 100.4 mAh·g^−1^ after 1800 cycles. The use of silica fume as a protective layer also represents a high-value application of industrial byproducts, offering new insights into anode design for aqueous zinc-ion batteries.

## Figures and Tables

**Table 1:** The content of components in silica fume (%).

Sample	SiO_2_	Fe_2_O_3_	K_2_O	CaO	Al_2_O_3_	Others
Unpurified material	90.18	2.64	1.93	1.42	1.67	2.16
After purification	95.43	1.43	1.07	0.48	0.41	1.18

## Data Availability

The original contributions presented in this study are included in the article. Further inquiries can be directed to the corresponding authors.

## Abbreviation

The following abbreviation is used in this manuscript:

SF	Silica Fume
